# Sustained delivery of PARP inhibitor Talazoparib for the treatment of *BRCA*-deficient ovarian cancer

**DOI:** 10.3389/fonc.2023.1175617

**Published:** 2023-05-09

**Authors:** Shicheng Yang, Allen Green, Needa Brown, Alexis Robinson, Merline Senat, Bryanna Testino, Daniela M. Dinulescu, Srinivas Sridhar

**Affiliations:** ^1^ Department of Chemical Engineering, Northeastern University, Boston, MA, United States; ^2^ Department of Pathology, Division of Women’s and Perinatal Pathology, Brigham and Women’s Hospital, Harvard Medical School, Boston, MA, United States; ^3^ Department of Radiation Oncology, Brigham and Women’s Hospital, Harvard Medical School, Boston, MA, United States; ^4^ Cancer Nanomedicine Co-ops for Undergraduate Research Experience (CaNCURE), Northeastern University, Boston, MA, United States; ^5^ Department of Physics, Northeastern University, Boston, MA, United States; ^6^ Department of Bioengineering, Northeastern University, Boston, MA, United States

**Keywords:** sustained delivery, PARP inhibitor, metastatic ovarian cancer, Talazoparib loaded implant, *BRCA* deficient mouse model

## Abstract

**Background:**

Ovarian cancer has long been known to be the deadliest cancer associated with the female reproductive system. More than 15% of ovarian cancer patients have a defective BRCA-mediated homologous recombination repair pathway that can be therapeutically targeted with PARP inhibitors (PARPi), such as Talazoparib (TLZ). The expansion of TLZ clinical approval beyond breast cancer has been hindered due to the highly potent systemic side effects resembling chemotherapeutics. Here we report the development of a novel TLZ-loaded PLGA implant (InCeT-TLZ) that sustainedly releases TLZ directly into the peritoneal (i.p.) cavity to treat patient-mimicking BRCA-mutated metastatic ovarian cancer (mOC).

**Methods:**

InCeT-TLZ was fabricated by dissolving TLZ and PLGA in chloroform, followed by extrusion and evaporation. Drug loading and release were confirmed by HPLC. The *in vivo* therapeutic efficacy of InCeT-TLZ was carried out in a murine *Brca2^-/-^p53^R172H/-^Pten^-/-^* genetically engineered peritoneally mOC model. Mice with tumors were divided into four groups: PBS i.p. injection, empty implant i.p. implantation, TLZ i.p. injection, and InCeT-TLZ i.p. implantation. Body weight was recorded three times weekly as an indicator of treatment tolerance and efficacy. Mice were sacrificed when the body weight increased by 50% of the initial weight.

**Results:**

Biodegradable InCeT-TLZ administered intraperitoneally releases 66 μg of TLZ over 25 days. *In vivo* experimentation shows doubled survival in the InCeT-TLZ treated group compared to control, and no significant signs of toxicity were visible histologically in the surrounding peritoneal organs, indicating that the sustained and local delivery of TLZ greatly maximized therapeutic efficacy and minimized severe clinical side effects. The treated animals eventually developed resistance to PARPi therapy and were sacrificed. To explore treatments to overcome resistance, *in vitro* studies with TLZ sensitive and resistant ascites-derived murine cell lines were carried out and demonstrated that ATR inhibitor and PI3K inhibitor could be used in combination with the InCeT-TLZ to overcome acquired PARPi resistance.

**Conclusion:**

Compared to intraperitoneal PARPi injection, the InCeT-TLZ better inhibits tumor growth, delays the ascites formation, and prolongs the overall survival of treated mice, which could be a promising therapy option that benefits thousands of women diagnosed with ovarian cancer.

## Introduction

Ovarian cancer is the second most common gynecologic cancer in USA, and is the deadliest cancer related to the female reproductive system. Data from the National Cancer Institute Surveillance, Epidemiology and End Results Program (NCI SEER) shows that in 2022, about 19,880 women were diagnosed with ovarian cancer with a 5-year relative survival of 49.7% (1). Most patients (57%) are diagnosed after peritoneal metastasis, leading to a 5-year survival of 30.8%. The high relapse rate (80%) of ovarian cancer requires additional secondary treatments, often leading to increased toxicity and eventual therapy resistance (2–6). Genetic inheritance plays an important role in ovarian cancer development, as specific genotypes can increase the likelihood of cancer development. Breast cancer genes 1 and 2 (*BRCA1, BRCA2*) are two tumor suppressor genes that strongly impact a women’s overall risk of developing breast and ovarian cancer (7–9). The resulting proteins, *BRCA1* and *BRCA2*, play a vital role in maintaining genomic stability and repairing DNA damage *via* homologous recombination (HR) (10, 11). On average, the risk of a woman developing ovarian cancer in her lifetime is 13% (12). However, women with pathogenic *BRCA1* or *BRCA2* mutations have a 55-72% and 45-69% chance of developing ovarian cancer by age 80, respectively (13–16). Population-based studies have shown that *BRCA1* and *BRCA2* mutations are found in roughly 15% of all ovarian cancer cases (17–19).

Poly (ADP-ribose) Polymerases (PARP) are a family of enzymes that are highly expressed in the nuclei of mammalian cells (20). PARP-mediated DNA repair is essential for survival in HR deficient *BRCA1/2* mutated cancer cells, making PARP an ideal therapeutic target (9, 21). PARP inhibitors (PARPi) are a class of anti-tumor drugs designed to block the action of PARP by inhibiting double-stranded break (DSB) repair pathways. Normal cells rely on high fidelity HR or low fidelity non-homologous end-joining DSB repair pathways. HR deficient malignancies, such as aggressive ovarian and breast cancers, are found to be highly sensitive to PARPi (22–24). This exploits the concept of synthetic lethality, as these cancer cells lose both ways to repair the DNA damage generated during replication. More than 50% of epithelial ovarian tumors have a defective HR DNA repair pathway, making these tumors prime candidates for PARPi (25). Several PARPi are clinically used to treat breast, ovarian, and prostate cancers (26). Olaparib was the first PARPi approved by the Food and Drug Administration (FDA) as a maintenance therapy for *BRCA*-mutated ovarian cancer in 2014 and received expanded authorization to treat fallopian tube and primary peritoneal tumors regardless of *BRCA* mutation status in 2017 (27, 28). Talazoparib (TLZ) is considered to be part of the next generation of PARPi and has about 100-fold higher lethality compared to first-generation drugs such as clinically approved Olaparib, Rucaparib, and Niraparib. Preclinical work has shown promise in TLZ-induced DNA damage regardless of *BRCA* mutations in ovarian and colon cancers thus expanding the therapeutic window for TLZ compared to other PARPi (29, 30). Although TLZ was approved for treatment of metastatic germline *BRCA1/2* breast cancer in 2018, expansion of approval to other malignancies has been hindered due to the more potent side effects associated with the drug (31, 32).

**Figure 1:**
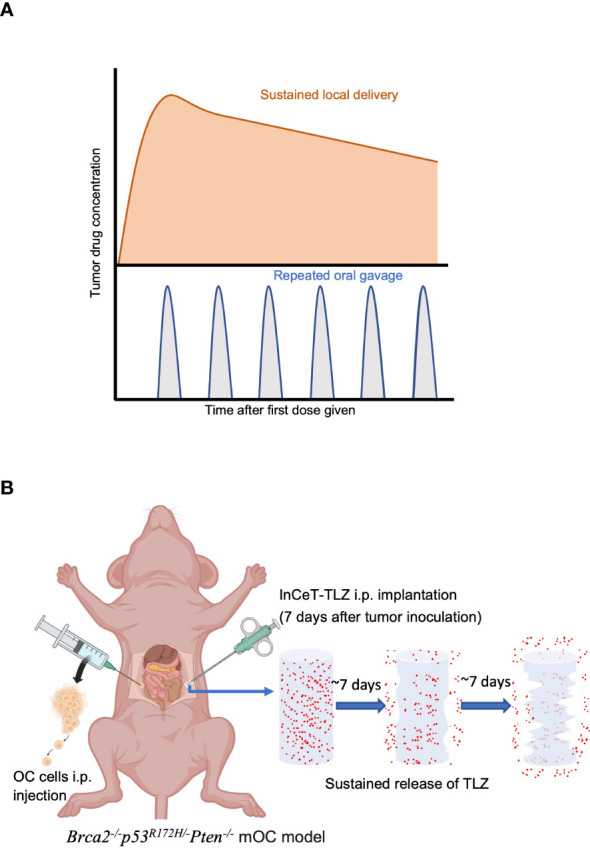
Sustained-release strategy maximizes therapeutic effect and minimizes toxicity. **(A)** Local sustained delivery optimizes drug accumulation at tumor site. **(B)** Schematic diagram of InCeT-TLZ (blue) progressively releasing TLZ (red) while degrading after being implanted into the i.p. cavity of *Brca2^-/-^p53^R172H/-^Pten^-/-^* mOC model.

For all therapeutic agents, the primary goal is to maintain a stable blood-drug concentration to achieve the maximum therapeutic effect while minimizing toxicity. PARPi are usually formulated as a daily oral pill (33, 34). Oftentimes, the blood-drug concentration associated with oral administration is affected by absorption, distribution, and elimination, which further depends on the hydrophobicity of the drug molecules, the residence time in the gastrointestinal tract, and the first pass effect related to liver degradation. The hydrophobic nature of TLZ molecules leads to low bioavailability when administered orally, which makes high dose usage necessary, thus leading to significant off-target toxicity (35). Due to its higher potency and ability to trap PARP, TLZ is recommended at a significantly lower dose of 1 mg daily compared to 300 mg or more for first-generation PARPi (31, 32). The half maximal inhibitory concentration (IC_50_) of TLZ is 0.57 nmol L^-1^ compared to 2 and 1.9 nmol L^-1^ for Olaparib and Rucaparib, respectively (36). In the EMBRACA clinical trial comparing 1 mg daily TLZ versus a conventional chemotherapy agent, adverse events were identified in 65% of patients receiving TLZ versus 50% in patients that received a chemotherapy agent. Of patients incurring adverse events, 53% required dose reduction below the therapeutic level due to either bone marrow toxicity, nausea, fatigue, or diarrhea (37). Therefore, developing novel drug delivery strategies to overcome systemic toxicity and increase tumor specific drug delivery is necessary. Sustained local delivery with degradable implants is considered a safer drug delivery method to achieve similar efficacy with lower dosage while minimizing off-target toxicity (38). Implants can be formulated so that the drug release rate results in a stable and sustained local drug concentration. The drug action period can be significantly extended even with just one implantation, and patient compliance can be improved due to reduced dosing frequency. Implants can be placed near or in the lesion site for local drug delivery, optimizing drug accumulation at the tumor site and decreasing off-target toxicity (**Figure 1A**). Sustained delivery is a promising strategy that helps drugs limited by their narrow therapeutic window or short blood-circulation to expand their medical applicability.

Although highly potent PARPi, such as TLZ, have shown promising results in cancer therapy for patients with *BRCA* mutations or HR deficiency, the development of resistance to PARPi in cancer cells severely limits the long-term survival of patients (39). Therefore, approaches to overcome resistance and prolong survival are required. Several mechanisms have been suggested for PARPi resistance, including reversion of *BRCA1/2* mutations and restoration of HR repair mechanisms (39). PARPi combined with drugs that impair HR repair, such as ATR inhibitors (ATRi), PI3K inhibitors (PI3Ki), CDK inhibitors (CDKi), or Wee1 inhibitors (Wee1i), is a potential strategy to overcome PARPi resistance. Evidence shows increased ATR-CHK1 activity with the development of PARPi resistance, and PARPi resistant cell lines have higher sensitivity to ATRi (40). Studies of PARPi with the PI3Ki Alpelisib (ALP), show that PI3K inhibition impairs HR pathways and enhances DNA damage accumulation (41, 42). The CDKi Dinaciclib (DCB), has been shown to ablate restored HR and reverse PARPi resistance (43–45). Wee1i, like Adavosertib, have shown promise in overcoming PARPi resistance by abrogating the G2/M checkpoint and inducing premature mitosis, thereby increasing DNA damage and sensitizing cancer cells to PARPi (46–50).

In this study, we formulated a sustained delivery TLZ implant (InCeT-TLZ) that delivers 66 μg of TLZ released over 25 days directly to the peritoneal cavity to treat patient-mimicking, *BRCA*-deficient metastatic ovarian cancer (mOC) (**Figure 1B**). The sustained release implant increased tumor specific drug delivery, improved survival compared to free TLZ, and minimized systemic toxicity that oftentimes leads to severe clinical side effects. Additionally, we tested several therapeutics using InCeT-TLZ treated ascites-derived cells to determine their feasibility in the treatment of PARPi resistant ovarian cancer lines. Our novel approach for sustained peritoneal delivery of TLZ offers a new alternative for *BRCA*-deficient mOC patients by substantially enhancing the therapeutic window and boosting cure rates, reducing mortality or suffering, and offering a major enhancement in quality of life for thousands of women diagnosed with ovarian cancer.

## Materials and methods

### InCeT-TLZ fabrication

InCeT-TLZ were fabricated using a polymer extrusion method as reported previously with further modifications (51, 52). Briefly, 10 mg of TLZ (AbMole BioScience, M1732, BMN637) and 180 mg poly lactic-co-glycolic acid (PLGA) (ALDRICH, BCCF0209, 7000-17000 Mw) were dissolved in 50 μl and 200 μl of chloroform, respectively. The two solutions were mixed with 5 min of sonication. The mixture was transferred to a 1 ml syringe and extruded into a silicone tubing (0.8 mm in diameter) at a flow rate of 2.5 μl/min. The tubes were kept in an oven at 60°C for 12 hours to dry and evaporate the chloroform. The dried tubes were then cooled to room temperature, cut into 5 mm segments, and stored at -20°C.

### InCeT-TLZ characterization

Drug loading capacity was tested by an Agilent high-performance liquid chromatography (HPLC) 1260 Infinity II system with SUPELCOSIL™ LC-18 hydrophobic alkyl phase HPLC column. Column dimensions were 15 cm x 3 mm with a particle size of 3 μm. Randomly selected InCeT-TLZ (n=3) with a recorded length of approximately 5 mm were dissolved in 100 μl of dichloromethane. Loaded TLZ was extracted by adding 900 μl of methanol followed by 5 min centrifugation. The supernatant was collected and diluted at 1:20 in mobile phase buffer. One microliter of each sample was injected for each run. HPLC was conducted using a gradient flow of 0.8 mL/min. The mobile phase contained 50% acetonitrile (containing 0.1% H_3_PO_4_) and 50% water (containing 0.1% H_3_PO_4_). The absorbance signal was measured by an Agilent 1260 Infinity II Diode Array Detector HS detection model at 232 nm with tret = 3.98 minutes. The standard curve of TLZ was built by dissolving weighted TLZ powder to appropriate concentrations at 1-100 μg/ml. A line of best fit was generated *via* linear regression and used to calculate the concentration of the InCeT-TLZ.

### Scanning electron microscopy

InCeT-TLZ was fractured with a cooled razor blade and attached to a specimen mount with a conductive carbon adhesive tab. It was sputter-coated with 10 nm of platinum using a CRESSINGTON Sputter Coater 108auto. Scanning electron microscope (SEM) images were produced in both secondary electrons and back-scattered electrons mode using a Hitachi S-4800 microscope at accelerating voltages of 3.0 kV and 5.0 kV with a current of 10 mA.

### Release kinetics studies

InCeT-TLZ were cut into 5 mm segments (n=3), placed into a 1.5 ml Eppendorf tube filled with 1 ml of PBS, and kept at 37°C to mimic biological conditions. The solution was replaced with fresh PBS buffer at each designated time point to maintain the concentration gradient and mimic the biological drug elimination. The removed buffer was labeled and kept at 4°C for HPLC analysis using the same parameters previously described to assess TLZ release.

### *In vitro* therapeutic efficacy

High grade serous ovarian cancer (HGSOC) cell lines were derived from the fallopian tubes of conditional *Brca2*; Tp53; Pten genetically engineered mouse models (mFT3666) (53). The mFT3666 lines were cultured in media consisting of 250 ml of DMEM/F12 (1:1) (Gibco, 11320) and 250 ml of M199 (Sigma, M4530) supplemented with 1.25 mg/ml of Bovine Serum Albumin (Sigma, A7030), 0.5 μg/mL of hydrocortisone, 25 ng/mL of Cholera Toxin, 25 ng/mL of Retinoic acid, 1% HEPES (1M) (gibco, 15630106), 20 mM L-glutamine, 1 ng/mL recombinant human EGF (Thermo Fisher Scientific, PHG0315), 1% Insulin-Transferrin-Selenium-Sodium Pyruvate (100X) (gibco, 51300044), and 1% Fetal Bovine Serum (Gibco, 10-438-034) (54, 55). The mFT3666 line was further transduced with a luciferase gene to monitor and analyze the *in vivo* tumor growth *via* IVIS bioluminescent imaging. Syngeneic *Brca1*; Tp53; Pten; Nf1; Myc genetically engineered mouse models (BPPNM) were also employed in this study (56). BPPNM lines were cultured in high glucose DMEM media (Thermo Fisher, 12430062) supplemented with 4% heat-inactivated fetal bovine serum (Gemini Bio, 100106), 1% penicillin-streptomycin (Thermo Fisher, 15140122), 1% insulin-transferrin-selenium (Thermo Fisher, 4140045), and 2 ng/mL of epithelial growth factor (Sigma Aldrich, E9644).

To evaluate the efficacy of InCeT-TLZ, mFT3666 cells were seeded into 96-well plates at a density of 500 cells per well. The following day, cells in each well were treated with InCeT-TLZ (1 mm or 2 mm) or TLZ in PBS solution (2.5 μg) or empty implant (2 mm). Cell viability was measured 7 days post treatment with an MTS assay (Promega). All experiments were done with n=6. A colony formation assay was also conducted to confirm treatment efficacy. The mFT3666 cells were seeded into a 6-well plate at a density of 1000 cells per well and treated with InCeT-TLZ (2 mm) or empty implant (2 mm). After a 7-day treatment, cells in each well were fixed with 3 ml of 10% neutral buffered formalin and then stained with 2 mL 0.01% (w/v) crystal violet. All treatments were done with n=4.

To screen for combinations of drugs to overcome PARPi resistance, ascites from BPPNM mice were collected and ascites-derived cell lines were established. Ascites cells were seeded into 96-well plates at a density of 1000 cells per well. Cells were treated with TLZ, CDKi (Dinaciclib; Selleckchem), ATRi (Ceralasertib; Selleckchem), or PI3Ki (Alpelisib; Selleckchem). Cell viability was measured 3-7 days post treatment with an MTS assay (Promega). IC_50_ values were calculated using nonlinear regression with normalized response using the GraphPad Prism 9 software. All experiments were done in triplicate.

### Western blot

Ovarian cancer (mFT3666; 500,000) cells were seeded on 10 cm plates and allowed to adhere overnight. The following day, 4 InCeT-TLZ (1mm) or empty implants (1mm) were scattered evenly across the plate. The plates were incubated for 3 days with daily agitation to ensure even dispersion of implants. After incubation, cells were lysed and the resulting cell lysate was stored at -80°C. 30 μg of cell lysate was loaded into a pre-cast 4%-20% protein gel (Biorad) and ran at 120V at room temperature. Following gel electrophoresis, proteins were transferred onto a PVDF membrane with a pore size of 0.2 μm (Immobilon) at 100V and 4°C for 1 hour. After transfer was complete, the membrane was rinsed with PBS and blocked for 1 hour with 5% dry milk at room temperature. The membrane was then incubated with its respective antibody (CST, 1:1000) diluted in the dry milk-based blocking buffer overnight at 4°C. The following day, the membrane was rinsed three times with PBS and incubated with an HRP-conjugated mouse anti-rabbit secondary antibody (Santa Cruz; 1:1000) for 1 hour at room temperature. After incubation, the membrane was washed three more times with PBS, incubated in 10 mL of SuperSignal West Pico Plus Chemiluminescent Substrate (Thermo Fisher) for 5 minutes, and imaged on a ChemiDoc XRS+ (Biorad).

### *In vivo* therapeutic efficacy

The *in vivo* therapeutic efficacy of InCeT-TLZ was carried out in a *Brca*; Tp53; Pten genetically engineered mOC mouse model, which was generated by injecting 5 million mFT3666 cells into the intraperitoneal (i.p.) cavity of a NCr Nude *nu/nu* (Charles River) mouse. All mice were monitored by IVIS imaging once a week to confirm tumor growth. On day 8 post-tumor inoculation, mice with normal-sized (4-5 mm in diameter) tumors were divided into 4 groups: PBS i.p. injection (n=3), empty implant i.p. implantation (n=4), TLZ i.p. injection (n=8), and InCeT-TLZ i.p. implantation (n=8). Mice in the i.p. TLZ treated group were given 0.33 mg/kg of TLZ by i.p. injection three times a week, while mice in the InCeT-TLZ treated group were implanted with InCeT-TLZ containing the same amount (2.5 mm, equal to a total amount of 10 treatments) of TLZ and a new InCeT-TLZ was implanted every 25 days. All mice were monitored by IVIS imaging once a week for luminescence to track tumor growth. The body weight of all mice was recorded three times a week. Mice were sacrificed when the body weight increased by 50% of the beginning weight. Organs (liver, spleen, kidney) and tumor were weighted and collected for histology. The growth of the tumor was represented by the luminescence intensity quantified from IVIS images. All animal studies and procedures were conducted under the Institutional Animal Care and Use Committee (IACUC) protocol #19-1240R reviewed and approved by the Northeastern University Institutional Animal Care and Use Committee (NU-IACUC).

A separate *in vivo* experiment was conducted to develop ascites-derived cell lines to study the effect of InCeT-TLZ on PARPi resistance acquisition. Syngeneic BPPNM cells (5x10^6^) were injected i.p. into 6-week-old immunocompetent C57BL/6J mice (Jackson Labs). Mice were divided equally into 4 experimental groups: PBS control; empty implant; 0.5 mg/kg i.p. TLZ three times a week; and InCeT-TLZ equivalent to 10 doses of i.p. TLZ. After 4 days, mice were treated with their respective treatments. Body weight was recorded three times a week. All mice developed ascites and were sacrificed once their body weight increased by 20% of their initial weight. Upon sacrifice, ascites was collected, and red blood cells were lysed with ACK lysis buffer. Once all red blood cells were lysed, the remaining ascites were plated on 10 cm plates and cultured in BPPNM media. All animal studies and procedures were conducted under IACUC protocol #2016N000212 and approved by the Brigham and Women’s Hospital IACUC Committee.

### Statistical analysis

Results within groups are presented as mean value and the standard error of mean. Results amongst groups were analyzed with a one-way ANOVA to test for significance (p<0.05) followed by a Tukey’s multiple comparison. Kaplan Meier method is used for survival analysis. Graphpad Prism 9 is used to do the statitstical analysis. All experiments were performed with mycoplasma-free cells.

## Results

### InCeT-TLZ fabrication and characterization

**Figure 2:**
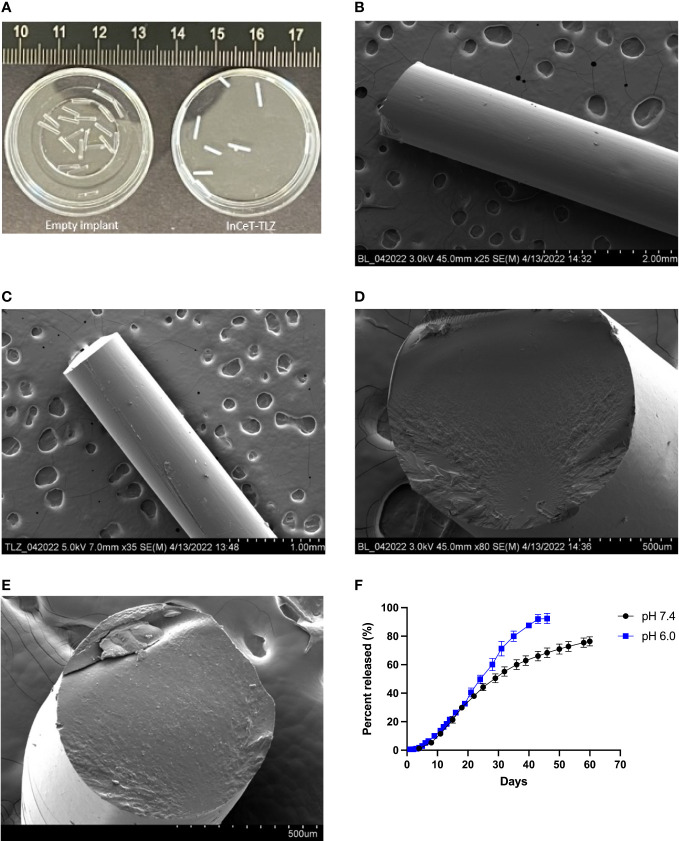
InCeT-TLZ formulation and characterization. **(A)** Photo of empty implant and InCeT-TLZ. SEM image top view of **(B)** empty implant and **(C)** InCeT-TLZ. SEM image cross-section of **(D)** empty implant and **(E)** InCeT-TLZ. **(F)** *In vitro* release profile of TLZ from 5 mm InCeT-TLZ (n=3) in PBS (pH 6.0 and 7.4) at 37°C.

HPLC results show that the actual TLZ loading of InCeT-TLZ is 26.75 μg/mm with an RT (Retention Time) of 4.1 minutes (**Figure S1**). InCeT-TLZ can be tailored to the desired drug dose by changing its overall length. The fabricated InCeT-TLZ is a light-yellow cylindrical rod with a diameter of 0.8 mm, allowing it to be placed in an 18-gauge needle for implantation (**Figures 2A**, **S2**). The InCeT-TLZ is stable over 6 months when kept at room temperature and can be kept at -20 °C for over two years.

Photos of empty implant and InCeT-TLZ are shown in **Figure 2A**. SEM figures show that both InCeT-TLZ and the empty implant have a smooth outer surface (**Figures 2B, C**). The empty implant has a smooth cross-section surface (**Figure 2D**), while the InCeT-TLZ has rough textures caused by the embedded TLZ microparticles (**Figure 2E**).

### InCeT-TLZ kinetic release study

To better understand how InCeT-TLZ dissolves within biological systems or tumor microenvironment, kinetic release studies were conducted at a pH of 7.4, normal physiological pH, and a pH of 6.0, tumor microenvironment pH (**Figure 2F**) (57, 58). The release results show that InCeT-TLZ can achieve sustained release with 80% of TLZ released from InCeT-TLZ with near zero-order release kinetics at both pH levels.

### InCeT-TLZ *in vitro* treatment efficacy

**Figure 3:**
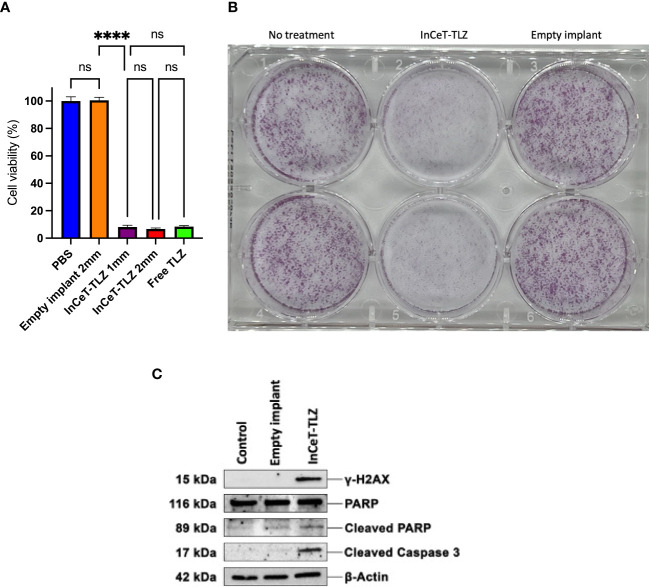
InCeT-TLZ enhances DNA damage and induces cell death in BRCA-deficient mFT3666 ovarian cancer cells. **(A)** Cell viability measured 7-day post treatment with an MTS assay (Promega). mFT3666 cells in 96-well plates were treated with InCeT-TLZ, TLZ in PBS solution, or empty implant. **(B)** Colony formation of mFT3666 cells in 6-well plate treated with InCeT-TLZ or empty implant. After a 7-day treatment, cells in each well were fixed and then stained with crystal violet. **(C)** Western blot analysis of untreated, empty implant treated, and InCeT-TLZ treated mFT3666 cells. mFT3666 cells were lysed after a 72-hours treatment with empty implant or InCeT-TLZ. DNA damage marker (γ-H2AX) and apoptotic markers (cleaved caspase 3 and cleaved-PARP) were detected via western blot. ****, p<0.0001. ns, not significant.

Cell viability assay results show that the empty implant treated group maintained 100% cell viability while the InCeT-TLZ treated groups showed roughly 7% viability in 96-well plates after a 7-day treatment (**Figure 3A**). In the 6-well plates, InCeT-TLZ treated wells showed significantly less and smaller colony formation compared to the control or empty implant treated groups, which corroborated these results (**Figure 3B**). *In vitro* treatment of the mFT3666 cell line with InCeT-TLZ resulted in increased expression of key markers related to PARP inhibition (**Figure 3C**). An increase in cleaved PARP was observed in the InCeT-TLZ treated sample. Additionally, increased expression of cleaved caspase 3, an apoptotic marker, and γ-H2AX, a marker of DNA damage, were seen after InCeT-TLZ treatment and no increase was observed in empty implant treated groups, confirming that increased cellular damage is due to TLZ release. These results demonstrate the implant’s efficacy as a novel PARP inhibitor formulation.

### InCeT-TLZ *in vivo* efficacy

**Figure 4:**
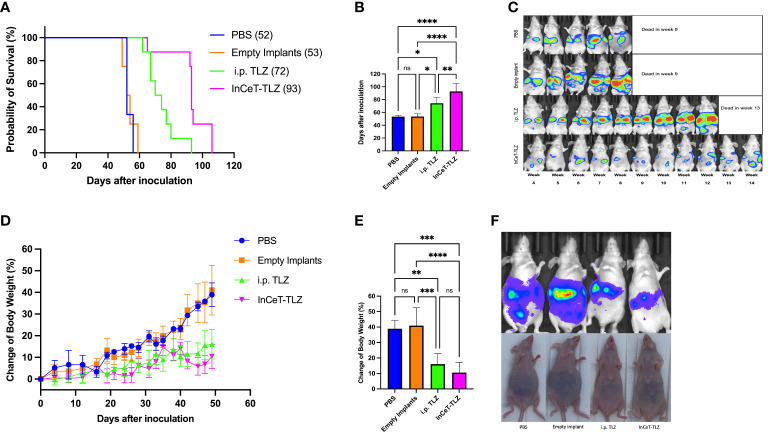
InCeT-TLZ inhibits tumor growth, delays ascites accumulation, prolongs survival in BRCA-deficient mOC model, and enhances the therapeutic efficacy of TLZ. Brca; Tp53; Pten genetically engineered mOC mice were divided into 4 groups and treated with PBS i.p. injection, empty implant i.p. implantation, TLZ i.p. injection, or InCeT-TLZ i.p. implantation 7 days after tumor inoculation. **(A)** Kaplan-Meier survival curve of mice engrafted with the mFT3666 cell line. Median survival reported in parentheses **(B)** Mean survival time of each group. **(C)** Luminescence image of randomly selected mice from each group. **(D)** Change in body weight from tumor engraftment up to day 49. **(E)** Mean value of change in body weight from tumor engraftment to day 49. **(F)** IVIS image with matched photo of randomly selected mice from each group on day 46. Kaplan-Meier method was used to analyze the significance of survival. One-way ANOVA and Tukey’s multiple comparison was used to test for significance. *, p<0.05; **, p<0.01; ***, p<0.001; ****, p<0.0001.

The survival curve showed that mice in InCeT-TLZ treated group survive longer than other groups (**Figure 4A**). Statistical analysis demonstrated that PBS and empty implant treated mice showed no significant difference in mean survival time. Mice treated with i.p. TLZ lived significantly longer with an average survival benefit of roughly 20 days compared to control. Mice implanted with InCeT-TLZ showed an even more substantial survival benefit, surviving an additional 20 days on average compared to i.p. TLZ treated mice (**Figure 4B**). Longitudinal IVIS imaging corroborated the survival results as seen in representative bioluminescent images of engrafted mFT3666 tumor growth (**Figure 4C**). Mice treated with TLZ had more delayed tumor development than mice treated with PBS or empty implants. Additionally, mice implanted with InCeT-TLZ showed even further delays in tumor development compared to their i.p. TLZ treated counterparts.

Consistent with the survival data, mice treated with PBS or empty implants do not show a clear difference in body weight change during the first 49 days (**Figure 4D**). Both control groups experienced a 40% increase in body weight due to ascites development by day 49, which is significantly faster than the two TLZ-treated groups (**Figure 4E**). Conversely, mice treated with i.p. TLZ or InCeT-TLZ only saw a roughly 10-16% increase in body weight during the same time period (**Figures 4D, E**). The body weight of mice from the two control groups increased gradually after tumor engraftment. However, mice in both TLZ-treated groups maintained a stable body weight in the first 20 days and a much slower increase thereafter. Tukey’s multiple comparison test showed no difference between the body weight progression of PBS treated group and the empty implant treated group, as well as no difference between the i.p. TLZ and InCeT-TLZ groups. **Figure 4F** depicts one randomly selected mouse from each group on day 46. Mice from the PBS and empty implant groups began to develop noticeable ascites by day 46, while mice from the i.p. TLZ group and InCeT-TLZ group still looked normal during the same time period. No significant morphology distortion was found for mice from any treatment groups compared to healthy mice *via* H&E staining (**Figure S3**).

### PARPi resistance acquisition and treatment

**Figure 5:**
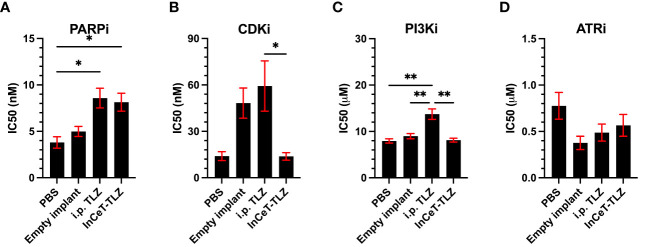
Drug resistance assays of ascites-derived cell lines. Calculated IC_50_ values after **(A)** PARPi, **(B)** CDKi, **(C)** PI3Ki, and **(D)** ATRi treatment. One-way ANOVA and Tukey’s multiple comparison was used to test for significance (*, p<0.05; **, p<0.01).

Recently, there has been a strong focus on overcoming the high prevalence of PARPi resistance acquisition in patients (59). Given our previous success with minimizing resistance acquisition using alternative formulations of PARPi, we developed 10 ascites-derived cell lines to determine the degree of PARPi resistance developed from each *in vivo* treatment and identify potential methods to overcome said resistance (60). As expected, cells derived from i.p. TLZ or InCeT-TLZ treated mice developed PARPi resistance, while cells derived from PBS or empty implants treated mice were still sensitive to PARPi treatment (**Figure 5A**). After validating that the TLZ-treated cell lines had developed resistance, we identified three potential therapies that are currently being explored to overcome PARPi resistance (42, 43, 61). When treated with Dinaciclib (CDKi), neither TLZ resistant condition showed significant changes in IC_50_ compared to control (**Figure 5B**). Interestingly, we observed a significant difference in CDKi susceptibility between the different PARPi administration methods. Cell lines previously treated with intraperitoneal injections of TLZ were found to be more resistant to CDKi than cell lines previously treated with InCeT-TLZ. Treatment with Alpelisib (PI3Ki) showed a significantly higher IC_50_ in the i.p. TLZ treated cells compared to all other treatment groups (**Figure 5C**). Most notably, all cell lines responded equally well to treatment with Ceralasertib (ATRi) with no significant changes in IC_50_ between treatment groups (**Figure 5D**).

## Discussion

The implant was designed to fit the widely available 18-gauge clinical needles for injection. Through the manufacturing process, InCeT-TLZ can be designed into various shapes to work with any specific implantation instruments or conditions. The dose of TLZ can be adjusted by changing the length of the InCeT-TLZ segments administered and can be further modified by the TLZ/PLGA ratio during fabrication for larger magnitude changes to address different purposes. Unlike the TLZ liquid solution or its nanoparticle suspension, TLZ stays in a solid state after being loaded in the PLGA-based implant, making InCeT-TLZ more stable and thus providing an extended storage period compared to its liquid formulations.

The faster release of InCeT-TLZ at pH 6.0 compared to pH 7.4 could be caused by the more robust degradation of the polymer’s glycolic acid and lactic acid bonds in a more acidic condition. Given the difference in tumor microenvironment and physiological pH, the change in release rate is further advantageous for targeting tumors, as the more acidic tumor microenvironment is capable of releasing TLZ at a faster rate due to this degradation (57, 58). The biocompatible and biodegradable nature of PLGA makes the implant safe for animal or human administration and eliminates the need to surgically extract the implant after treatment.

The *in vitro* InCeT-TLZ treatment results indicate that a sufficient amount of TLZ is released from the InCeT-TLZ to effectively inhibit PARP and cause cell death in ovarian cancer cells during the 7-day treatment period. The 1 mm and 2 mm InCeT-TLZ treated cells did not show a significant change in viability between groups, indicating that even a singular 1 mm InCeT- TLZ at a dose of only 26.75 μg/mm is potent enough to cause widespread cell death *in vitro*. Based on the release kinetics curve, approximately 6% of TLZ was released from InCeT-TLZ during the treatment period, which is 1.61mg and 3.21mg for the 1 mm and 2 mm InCeT-TLZ, respectively. The final concentration would be 21.16 mM or 42.32 mM in a 96- well plate for 1 mm or 2 mm InCeT-TLZ treated wells, which is much more than enough to kill most of the tumor cells considering the IC_50_ of TLZ is around 10 nM for this cell line (60). The 7% of cancer cells remaining after TLZ treatments could be caused by acquired resistance, which is an expected challenge to overcome in future iterations of InCeT-TLZ. The western blot results revealed DNA damage caused by the *in vitro* InCeT-TLZ treatments and confirmed its effectiveness to this cell line.

Previous experiments and literature have shown that many late-stage ovarian cancer mouse models develop ascites within the intraperitoneal cavity (60, 62), which can be reflected by increases in body weight. Therefore, in this study, the endpoint was set at a 50% increase in body weight. The faster body weight increase of the two control groups indicated that the tumor progression and ascites development within these mice was much faster than mice in the TLZ-treated conditions. These results were further supported by IVIS imaging which depicted more pronounced abdominal swelling due to ascites accumulation as the tumors progressed. Furthermore, ascites could be observed through the skin after day 30 for mice from the two control groups, while it was delayed to day 60 or day 80 for the TLZ or InCeT-TLZ treated groups, respectively, meaning that InCeT-TLZ treatment significantly inhibited tumor growth and delayed ascites generation. Survival curves demonstrated an improvement in the therapeutic efficacy of InCeT-TLZ compared to TLZ i.p. injection. Mice in these two groups received equal average doses of TLZ, however, the InCeT-TLZ treated group showed a more prolonged survival and a significantly longer average lifespan than the TLZ i.p. injection treated group. This indicates that the implant provided a slower and more stable local release of TLZ in the peritoneal cavity, maintaining a near-fixed TLZ concentration to provide a consistent therapeutic effect, maximizing the disease site dose accumulation while minimizing off-target toxicity. On the contrary, traditional oral gavage and injections involve drug absorption, distribution, and elimination, leading to a considerable fluctuation of the blood-drug concentration between two or more doses. Furthermore, when administering TLZ *via* gavage or injection, the blood-drug concentration can drop below the minimum effective level or increase above the toxic concentration level, leading to ineffective or harmful responses. The sustained-local release implant could solve these problems by gradually releasing drug directly into the tumor microenvironment, which greatly reduces the amount of drug entering blood circulation to minimize any systematic toxicity. Moreover, the sustained release implant also has important implications in patients with prior ileostomies, which impair PARPi absorption. Similarly, malignant bowel obstruction occurs in 50% patients with advanced ovarian cancer (63), and they will not be able to swallow chemotherapy pills, making the sustained delivery implants beneficial. Thus, using the sustained release implant, patients could have an increased therapeutic effect with decreased off-target side effects with the same amount of drug given compared to traditional drug administration methods. The medical application of drugs such as TLZ and DCB are greatly limited by their toxicity (64, 65). Our sustained delivery strategy can potentially broaden the medical scope of these drugs and give physicians the option to prescribe higher dosages to patients using this sustained local drug delivery method. Compared to traditional oral and i.p. injection of free drug or lipid formulations, our sustained drug delivery implants have several advantages: (i) drug loading is adjustable and is not greatly limited by its hydrophobicity; (ii) loading ratio and release profile can be modified during implant fabrication; (iii) higher tumor drug accumulation compared to systematic administration; (iv) lower systemic toxicity due to local delivery; and (v) better patient compliance with only 1 injection needed per month. One possible drawback of the sustained delivery implants is that the total dosage it carries is significantly higher than a single injection, which may lead to hematological toxicity if the release is accelerated in some cases. However, physicians could easily take those implants out with a biopsy needle to stop the release when toxicity is observed. Another consideration for the local i.p. sustained delivery of TLZ is the potential for remote metastasis, particularly in the CNS. Brain metastasis in epithelial ovarian cancer is relatively rare, with an incidence of 1.17% (66, 67); however, the risk increases threefold to 3.84% (19) in the presence of BRCA mutations. This highlights the importance of ensuring the systemic efficacy of this i.p. local delivery of TLZ. Although the majority of the drug is released and absorbed locally, a certain portion should also be distributed systemically through blood vessels. Future pharmacodynamic and pharmacokinetic studies are necessary to confirm this and provide a comprehensive understanding of the treatment’s systemic effects.

Similar to i.p. TLZ, InCeT-TLZ still resulted in acquired PARPi resistance in murine models. However, cells treated with InCeT-TLZ appear to respond differently to other therapies that are currently being explored as alternative treatments to overcome PARPi resistance (43, 59, 60). PARPi i.p. treatment resulted in increased resistance to CDKi when compared to InCeT-TLZ treatment. Additionally, i.p. PARPi treatment also resulted in increased resistance to PI3Ki compared to all other treatment conditions. These results suggest that the InCeT-TLZ can potentially minimize cross-resistance to other therapies and lead to more effective treatment in patients who have developed PARPi resistance. ATRi treatment showed no significant differences in efficacy in any treatment group, demonstrating its effectiveness as a second-line therapy in patients who develop PARPi resistance. In addition to exhibiting the potential for these therapies to function as second-line treatments, these results also highlight the potential for exploring InCeT formulations for these inhibitors as single-therapy implants or combination implants with PARPi. By expanding the InCeT implant platform with these novel therapeutics, we hope to develop more efficacious and less toxic treatments similar to the InCeT-TLZ showcased here.

## Conclusion

In this study, a TLZ-loaded PLGA implant was developed for the sustained and local delivery of TLZ to treat high-grade serous ovarian cancer. The TLZ loading of InCeT-TLZ was 26.75 μg/mm, and it can be implanted into a human or animal easily with an 18-gauge needle. In animal studies, the extended survival of InCeT-TLZ treated mice indicated that the slow release of TLZ maximizes PARP inhibitor therapy in the peritoneal cavity for disseminated cancer treatment. Additionally, the ability to decrease ascites formation with the intraperitoneal InCeT-TLZ suggests it can be a promising treatment for ovarian cancer-associated ascites and disease progression. Histopathology following peritoneal InCeT-TLZ implantation showed no toxicity in any surrounding organs at risk. The therapeutic efficacy of the ATR inhibitor and the PI3K inhibitor on ascites cell lines from TLZ resistant BPPNM mice showed that the IC_50_ was comparable to non-resistant cell lines, suggesting the potential for future combination therapies. This approach would offer new sustained therapy options for substantially enhancing the therapeutic window, increasing cure rates, reducing mortality and suffering, and offering major enhancements in quality of life for the thousands of women diagnosed with OC yearly.

## Data availability statement

The raw data supporting the conclusions of this article will be made available by the authors, without undue reservation.

## Ethics statement

The animal study was reviewed and approved by Northeastern University Institutional Animal Care and Use Committee; Brigham and Women’s Hospital Institutional Animal Care and Use Committee.

## Author contributions

SY contributed to the whole project and drafted this manuscript. AR, MS, and BT contributed to the *in vivo* experiments. AG contributed and drafted the combination treatment part. NB contributed to the data interpretation and made critical revision of this manuscript. SS and DD conceived and supervised the project and are the senior advisors. The work reported in the paper has been performed by the authors, unless clearly specified in the text. All authors contributed to the article and approved the submitted version.
