# Supplementary material for: Sustained delivery of PARP inhibitor Talazoparib for the treatment of BRCA-deficient ovarian cancer
Source: Front Oncol. 2023 May 9;13:1175617. doi: 10.3389/fonc.2023.1175617 (PMC10203577; doi:10.3389/fonc.2023.1175617)
Supplement: Supplementary file 1 [file DataSheet_1.docx]

**Supplementary Material**


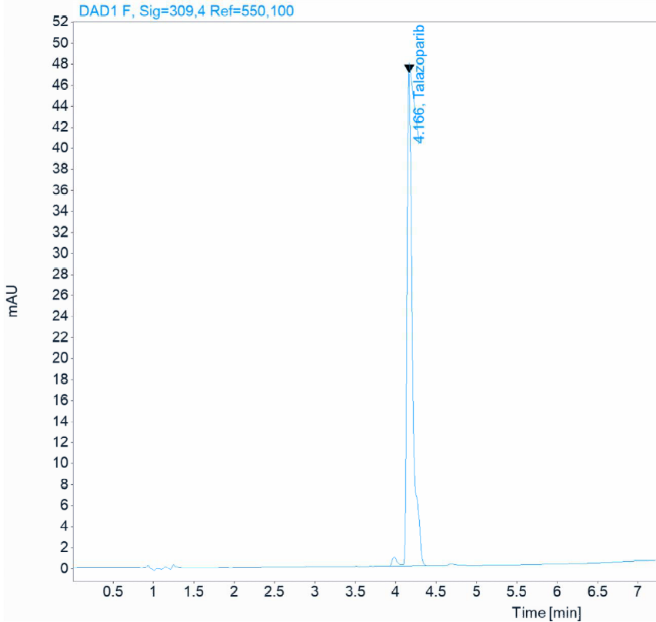


Figure S1. HPLC result of TLZ encapsulated in InCeT-TLZ


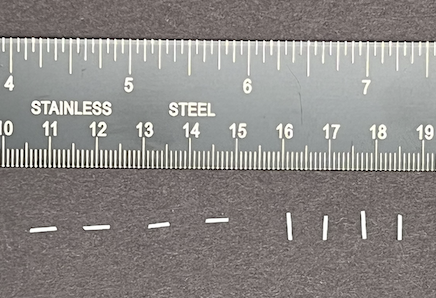

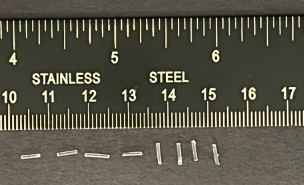


Empty implant

InCeT-TLZ

Figure S2. Photo of empty implant and InCeT-TLZ.


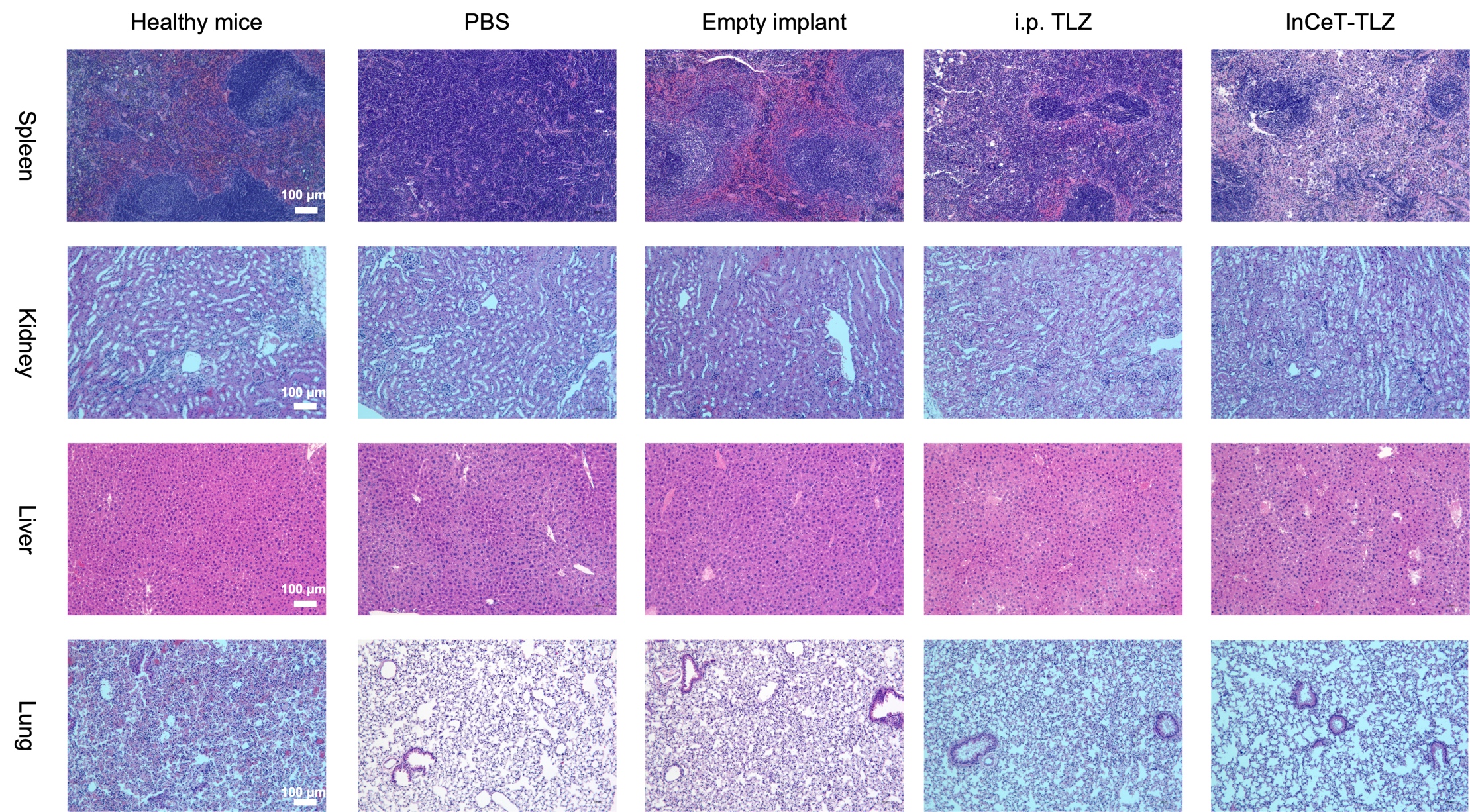


Figure S3. H&E staining of spleen, kidney, liver, and lung from healthy mice and mice from the four treatment groups with mOC generated with the mFT3666 cell line. No significant morphological differences were observed.
